# Topological and organizational properties of the products of house-keeping and tissue-specific genes in protein-protein interaction networks

**DOI:** 10.1186/1752-0509-3-32

**Published:** 2009-03-11

**Authors:** Wen-hsien Lin, Wei-chung Liu, Ming-jing Hwang

**Affiliations:** 1 Institute of Biomedical Informatics, National Yang-Ming University, Taipei 112, Taiwan; 2 Institute of Biomedical Sicences, Academia Sinica, Taipei 115, Taiwan; 3 Institute of Statistical Science, Academia Sinica, Taipei 115, Taiwan

## Abstract

**Background:**

Human cells of various tissue types differ greatly in morphology despite having the same set of genetic information. Some genes are expressed in all cell types to perform house-keeping functions, while some are selectively expressed to perform tissue-specific functions. In this study, we wished to elucidate how proteins encoded by human house-keeping genes and tissue-specific genes are organized in human protein-protein interaction networks. We constructed protein-protein interaction networks for different tissue types using two gene expression datasets and one protein-protein interaction database. We then calculated three network indices of topological importance, the degree, closeness, and betweenness centralities, to measure the network position of proteins encoded by house-keeping and tissue-specific genes, and quantified their local connectivity structure.

**Results:**

Compared to a random selection of proteins, house-keeping gene-encoded proteins tended to have a greater number of directly interacting neighbors and occupy network positions in several shortest paths of interaction between protein pairs, whereas tissue-specific gene-encoded proteins did not. In addition, house-keeping gene-encoded proteins tended to connect with other house-keeping gene-encoded proteins in all tissue types, whereas tissue-specific gene-encoded proteins also tended to connect with other tissue-specific gene-encoded proteins, but only in approximately half of the tissue types examined.

**Conclusion:**

Our analysis showed that house-keeping gene-encoded proteins tend to occupy important network positions, while those encoded by tissue-specific genes do not. The biological implications of our findings were discussed and we proposed a hypothesis regarding how cells organize their protein tools in protein-protein interaction networks. Our results led us to speculate that house-keeping gene-encoded proteins might form a core in human protein-protein interaction networks, while clusters of tissue-specific gene-encoded proteins are attached to the core at more peripheral positions of the networks.

## Background

One of the major aims in modern molecular biology is to identify how living organisms are brought into existence from the basic building blocks of life, such as genes and their protein products. With the completion of the human genome project and recent advances in molecular biology, a complete understanding of the chromosomal organization of human genes will become possible in the not so distant future [1]. In the post-genomic era, the next step in modern molecular biology is to understand how gene products, or proteins, interact to perform cellular functions [2]. The human body is composed of millions of cells which differ greatly in morphology despite the fact that they all possess the same set of genetic information. Some genes are persistently transcribed and expressed in all cells and are called house-keeping genes, as they are involved in the basic cellular functions required for the maintenance of a cell. For instance, the genes that code for histones, proteins responsible for DNA packaging in chromatin [3], are universally expressed in all cells [4]. Other genes are expressed only in cells of certain tissue types, also known as tissue-specific genes, and are thought to be responsible for the cell diversity observed in living organisms today. A good example is found in the immune system, where the human leukocyte antigen genes and their regulatory proteins are specifically expressed in macrophages and B cells [5]. Using microarray-based [6] and tag-based [7,8] techniques, gene expression patterns in different tissue types can be easily quantified, and the identification of house-keeping and tissue-specific genes is possible with modern statistical analysis [4,9]. However, little is known about how the protein products of house-keeping and tissue-specific genes are organized or embedded within the protein-protein interaction (PPI) networks that ultimately give rise to the observed similarities and differences in morphology between cells. In this paper, we employed the tool of network analysis to address this issue.

Network analysis has its origin in sociology, but, in recent years, has been successfully applied to different fields of biological sciences from molecular biology, proteomics, medicine to ecology and epidemiology [10-18]. A major goal of network analysis is to reveal the structural organization of a network and propose mechanisms that may give rise to the observed network topology [11,19]. For instance, the nodal connection of several biological networks tends to follow a power law distribution, with the majority of nodes having only a small number of neighbors and only a few having many [10,20,21]. Such a power law distribution in connectivity renders a network robust against random attacks [11,22], and the preferential attachment model of network evolution has been proposed as a possible mechanism that gives rise to such a power law distribution [11,22]. Another goal of network analysis is to quantify or characterize the position of individual nodes in a network and relate this information to the biological roles in which they might be involved [10,14,17,18,23-26]. For instance, Jeong et al. [10] analyzed the yeast PPI network and found that genes coding for proteins that have many interacting partners tend to be essential genes vital to cell survival.

This study had two aims. The first was to use network analysis to elucidate the topological importance of house-keeping and tissue-specific genes (or, more precisely, their protein products) in human PPI networks by asking whether proteins encoded by house-keeping genes or tissue-specific genes tend to occupy topologically important positions or not. Topological importance here simply refers to how prominent or central a node is to others in the same network and can be measured in different ways. The second aim was, using an anthropomorphic analogy to humans who tend to arrange tools performing similar tasks in close vicinity to each other, to determine how nature organizes tools (house-keeping genes and tissue-specific genes) in human PPI networks. Specifically, we asked whether the protein products of house-keeping genes or tissue-specific genes tend to connect or interact among themselves in a PPI network; for convenience, we define such a connection pattern as homophylic connectivity. We examined these issues using two different datasets to test the robustness of our findings. The paper is organized as follows. We first describe the databases used and how the lists of house-keeping genes and tissue-specific genes were acquired, then how we constructed the different PPI networks. We then describe the three commonly-used measures of topological importance, how homophylic connectivity in a PPI network was quantified, and how the statistical significance of our findings was tested. Finally, we present the results and discuss their implications.

## Results

### Basic network statistics

The Human Gene Expression Index (HuGE Index) database [4] contains gene expression data for 19 different tissue types. For simplicity, we identify here each tissue-type by the name of the organ from which the tissue was derived. For each tissue type, we mapped the genes expressed to the Human Protein Reference Database (HPRD) [27] and identified the corresponding proteins in order to construct a tissue-specific PPI network. The HuGE Index database provides a list of expressed genes and a list of tissue-specific genes for each tissue type, together with a list of house-keeping genes expressed in all tissues. Here a node in a PPI network represents a protein, nodes representing proteins encoded by house-keeping genes are called house-keeping nodes and those representing proteins encoded by tissue-specific genes are tissue-specific nodes. Table 1 summarizes, for each tissue type, the total number of nodes in the PPI network and the number of house-keeping and tissue-specific nodes. We also constructed the EST-SAGE dataset (see Methods), which contains gene expression data for 20 different tissue types, and mapped the expressed genes to the HPRD and constructed the PPI networks for the different tissue types. Again, we identify each tissue-type by the name of the organ from which the tissue was derived. Table 2 summarizes, for each tissue type, the total number of nodes and the number of house-keeping and tissue-specific nodes in the corresponding PPI network. The network data for different tissue types are given in additional file 1. Tables 1 and 2 also provide the proportions of house-keeping nodes and tissue-specific nodes in the total number of nodes for each tissue type. The proportion of house-keeping nodes varied from 0.105 to 0.321 for the HuGE Index dataset and from 0.051 to 0.224 for the EST-SAGE dataset, while the corresponding values for the proportion of tissue-specific nodes were 0.017 to 0.173 and 0.008 to 0.080. With a few exceptions, each PPI network tended to have more house-keeping nodes than tissue-specific nodes. Furthermore, each PPI network consists of a large network fragment (a fragment contains nodes that are only reachable from those in the same fragment) and several much smaller fragments (Tables 3 and 4). The proportion of total number of nodes in the largest network fragment varied from 0.901 to 0.975 among different PPI networks for the HuGE Index dataset and from 0.831 to 0.970 for the EST-SAGE dataset; therefore the extent of connectivity of every PPI network constructed in this study is high.

**Table 1:** A summary of numbers of nodes in HuGE Index-derived PPI networks.

Tissue	Total number of nodes	Number of house-keeping nodes	Number of tissue-specific nodes
Blood	907	179 (0.197)	59 (0.065)
Brain	1048	184 (0.176)	121 (0.115)
Breast	466	148 (0.318)	18 (0.039)
Cervix	934	184 (0.197)	26 (0.028)
Colon	684	176 (0.257)	13 (0.019)
Endometrium	1209	194 (0.160)	65 (0.054)
Esophagus	996	185 (0.186)	46 (0.046)
Kidney	1774	200 (0.113)	263 (0.148)
Liver	1646	200 (0.122)	244 (0.148)
Lung	1933	203 (0.105)	318 (0.165)
Muscle	1611	194 (0.120)	262 (0.163)
Myometrium	1112	190 (0.171)	46 (0.041)
Ovary	1239	190 (0.153)	108 (0.087)
Placenta	896	176 (0.196)	44 (0.049)
Prostate	1917	202 (0.105)	331 (0.173)
Spleen	605	160 (0.264)	13 (0.021)
Stomach	516	159 (0.308)	9 (0.017)
Testes	464	149 (0.321)	8 (0.017)
Vulva	1270	193 (0.152)	65 (0.051)

The figures in parenthesis are the proportion of house-keeping nodes and the proportion of tissue-specific nodes in the total number of nodes in a particular PPI network.

**Table 2:** A summary of numbers of nodes in EST-SAGE-derived PPI networks.

Tissue	Total number of nodes	Number of house-keeping nodes	Number of tissue-specific nodes
Blood	3613	233 (0.062)	133 (0.037)
Blood vessel	1735	177 (0.102)	34 (0.020)
Bone marrow	2278	207 (0.091)	31 (0.014)
Brain	4624	238 (0.051)	368 (0.080)
Breast	3796	235 (0.062)	47 (0.012)
Colon	2057	186 (0.090)	54 (0.026)
Eye	4003	228 (0.057)	70 (0.017)
Heart	2035	188 (0.092)	33 (0.016)
Kidney	1388	167 (0.120)	17 (0.012)
Liver	1650	166 (0.101)	55 (0.033)
Lung	2624	198 (0.075)	30 (0.011)
Lymph node	438	98 (0.224)	15 (0.034)
Muscle	1974	192 (0.097)	65 (0.033)
Ovary	1316	164 (0.125)	10 (0.008)
Pancreas	2023	191 (0.094)	21 (0.010)
Placenta	3187	214 (0.067)	101 (0.032)
Prostate	3181	221 (0.069)	28 (0.009)
Skin	950	133 (0.140)	12 (0.013)
Stomach	2440	194 (0.080)	24 (0.010)
Thyroid gland	606	113 (0.186)	10 (0.017)

The figures in parenthesis are the proportion of house-keeping nodes and proportion of tissue-specific nodes.

**Table 3:** The distribution of network fragment size in HuGE Index-derived PPI networks.

Tissue	Distribution of network fragment size	Proportion of total number of nodes in the largest network fragment
Blood	848(1), 5(1), 3(1), 2(20)	0.948
Brain	998(1), 3(4), 2(19)	0.952
Breast	441(1), 3(1), 2(11)	0.946
Cervix	879(1), 3(7), 2(17)	0.941
Colon	643(1), 5(1), 4(2), 3(2), 2(11)	0.940
Endometrium	1162(1), 3(1), 2(22)	0.961
Esophagus	955(1), 5(1), 4(1), 2(16)	0.959
Kidney	1714(1), 4(1), 3(4), 2(22)	0.966
Liver	1590(1), 4(1), 3(4), 2(20)	0.966
Lung	1873(1), 3(4), 2(24)	0.969
Muscle	1570(1), 3(3), 2(16)	0.975
Myometrium	1062(1), 3(2), 2(22)	0.955
Ovary	1173(1), 3(4), 2(27)	0.947
Placenta	848(1), 5(1), 3(1), 2(20)	0.946
Prostate	1859(1), 3(6), 2(20)	0.970
Spleen	570(1), 4(3), 3(1), 2(10)	0.942
Stomach	470(1), 5(1), 4(1), 3(3), 2(14)	0.911
Testes	418(1), 4(2), 3(4), 2(13)	0.901
Vulva	1225(1), 3(3), 2(18)	0.965

In the middle column, each number represents the size of a network fragment and in the following parenthesis is the number of fragments belonging to this network fragment size.

**Table 4:** The distribution of network fragment size in EST-SAGE-derived PPI networks.

Tissue	Distribution of network fragment size	Proportion of total number of nodes in the largest network fragment
Blood	3493(1), 5(2), 3(8), 2(43)	0.967
Blood vessel	1658(1), 4(2), 3(5), 2(27)	0.956
Bone marrow	2173(1), 7(1), 4(1), 3(6), 2(38)	0.954
Brain	4477(1), 7(1), 5(2), 4(1), 3(6), 2(54)	0.968
Breast	3679(1), 5(2), 4(1), 3(7), 2(41)	0.969
Colon	1950(1), 4(3), 3(7), 2(37)	0.948
Eye	3853(1), 5(3), 4(2), 3(9), 2(50)	0.963
Heart	1935(1), 5(1), 4(3), 3(5), 2(34)	0.951
Kidney	1296(1), 8(1), 4(1), 3(4), 2(34)	0.934
Liver	1551(1), 5(1), 4(1), 3(8), 2(33)	0.940
Lung	2502(1), 5(2), 4(2), 3(6), 2(43)	0.954
Lymph node	364(1), 7(1), 6(1), 5(1), 3(4), 2(22)	0.831
Muscle	1878(1), 4(1), 3(8), 2(34)	0.951
Ovary	1246(1), 3(6), 2(26)	0.947
Pancreas	1920(1), 5(1), 4(1), 3(6), 2(38)	0.949
Placenta	3076(1), 6(1), 5(1), 3(8), 2(38)	0.965
Prostate	3062(1), 7(1), 5(1), 4(1), 3(7), 2(41)	0.963
Skin	891(1), 3(5), 2(22)	0.938
Stomach	2303(1), 4(1), 3(5), 2(59)	0.944
Thyroid gland	546(1), 6(1), 4(2), 3(2), 2(20)	0.901

In the middle column, each number represents the size of a network fragment and in the following parenthesis is the number of fragments belonging to this network fragment size.

The HuGE and EST-SAGE datasets have 12 tissue types in common (Table 5). For each of those common tissue types, we determined the number of nodes common to both PPI networks and the number of common house-keeping and tissue-specific nodes and determined the extent of overlap of nodes of a certain type between the two datasets. As shown in Table 5, depending on the tissue type considered, the percentage of common nodes (number of common nodes/total number of nodes × 100) in a HuGE-derived PPI network varied from 32.8% to 93.5%, while the percentage of common house-keeping nodes and common tissue-specific nodes varied from 8.75% to 31.3% and from 0% to 4.48%, respectively. Again depending on the tissue type considered, the percentage of common nodes in a EST-SAGE-derived PPI network varied from 11.3% to 49.6%, while the percentage of common house-keeping nodes and common tissue-specific nodes varied from 3.85% to 10.9% and from 0% to 1.02%, respectively. The extent of overlap between the two datasets was low, so it is reasonable to say that the results derived from one dataset can complement those derived from the other.

**Table 5:** Tissue types that appear in both the HuGE and the EST-SAGE datasets.

Tissue	Percentage of common nodes in the total number of nodes	Percentage of common house-keeping nodes	Percentage of common tissue-specific nodes
Blood	93.5%, 23.5%	18.7%, 4.71%	0.55%, 0.14%
Brain	92.9%, 21.1%	17.3%, 3.91%	4.48%, 1.02%
Breast	92.3%, 11.3%	31.3%, 3.85%	0%, 0%
Colon	64.3%, 21.4%	21.1%, 7.00%	0%, 0%
Kidney	38.8%, 49.6%	8.12%, 10.4%	0.28%, 0.36%
Liver	49.0%, 48.8%	8.75%, 8.73%	0.73%, 0.73%
Lung	61.0%, 44.9%	8.85%, 6.52%	0.21%, 0.15%
Muscle	53.6%, 43.7%	10.1%, 8.26%	1.18%, 0.96%
Ovary	44.6%, 42.0%	11.5%, 10.9%	0%, 0%
Placenta	82.7%, 23.3%	18.3%, 5.15%	0.67%, 0.19%
Prostate	71.4%, 43.0%	10.0%, 6.04%	0.10%, 0.06%
Stomach	72.7%, 15.4%	26.4%, 5.57%	0.19%, 0.04%

The first number for each entry is for the HuGE-derived PPI network and the second for the EST-SAGE-derived PPI network.

### Topological properties of the house-keeping nodes

**Figure 1:**
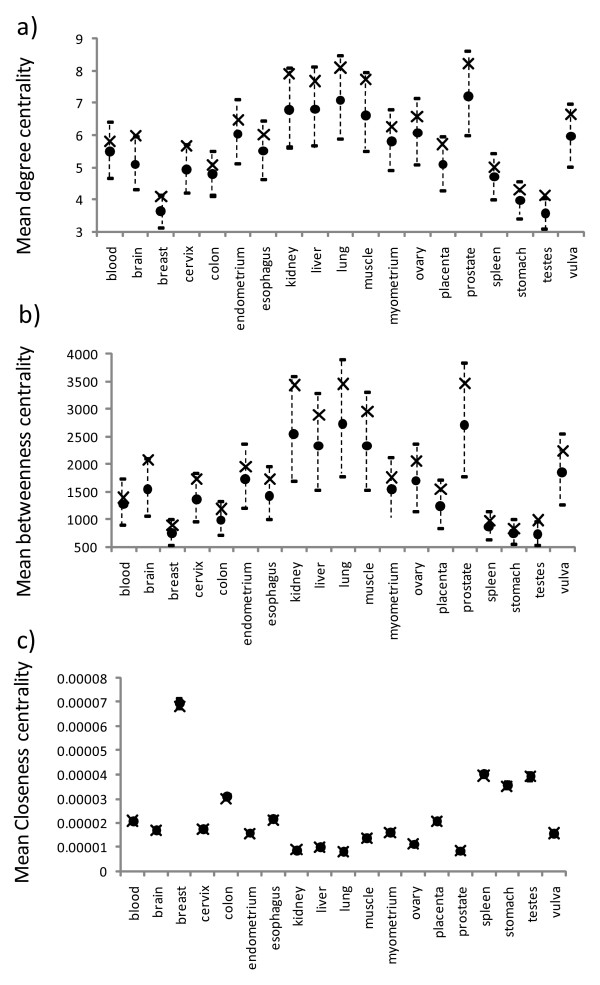
**Observed mean topological properties for the house-keeping nodes in different PPI networks for the HuGE Index dataset**. The mean degree, betweenness, and closeness centralities are shown in a), b), and c), respectively. Each observed mean is shown as a cross and each expected mean by a circle, with its 95% confidence intervals shown as a vertical dotted line above and below the expected mean.

**Figure 2:**
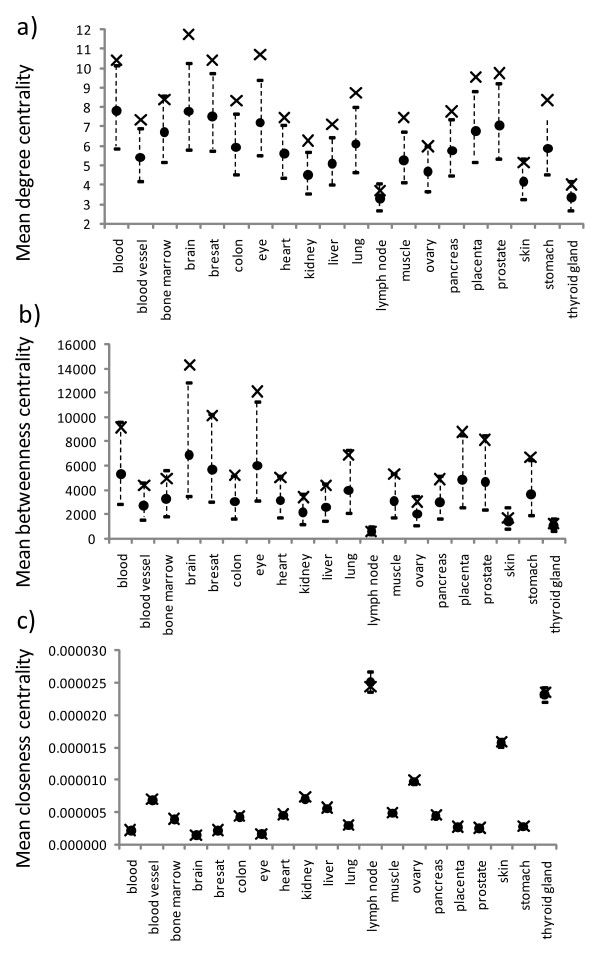
**Observed mean topological properties for the house-keeping nodes in different PPI networks for the EST-SAGE dataset**. The mean degree, betweenness, and closeness centralities are shown in a), b), and c), respectively. Each observed mean is shown as a cross and each expected mean by a circle, with its 95% confidence intervals shown as a vertical dotted line above and below the expected mean.

The topological importance of a node can be quantified using different centrality measures (see Methods). Those commonly used are the degree centrality (the number of direct neighbors of a node), the betweenness centrality (an index quantifying how frequently a node appears on all shortest paths between all node pairs) and the closeness centrality (an index measuring how close a node is to all others in the same network). For each PPI network, we determined these three centralities for all individual nodes, then picked out the house-keeping nodes and calculated their means. We also calculated the expected distribution of these means if the collection of house-keeping nodes were a random subset of nodes in the PPI network. Figures 1 and 2 summarize the results for the HuGE Index and EST-SAGE databases, respectively.

For the HuGE Index dataset, only the house-keeping nodes in the brain and testes tissues had observed mean degree centralities significantly greater than the expected means (the observed means are located well outside the 95% confidence intervals of the model distribution) (Figure 1a). Despite the non-significant results, the observed mean degree centralities for the remaining tissue types were all greater than the expected means (Figure 1a). For the betweenness centrality, only the house-keeping nodes in the testes tissue showed a significant difference from the expected mean despite all tissues having observed means greater than the expected means (Figure 1b). For the closeness centrality, none of the observed means were significantly different from the expected means, although eight tissues had observed means that were greater than the expected means, while eleven had observed means lower than expected (Figure 1c and additional file 2, Table S1).

All but four tissue types in the EST-SAGE dataset showed that the observed mean degree centralities for house-keeping nodes were significantly greater than the expected means (note that the observed means were all greater than the expected means for all tissue types) (Figure 2a). For the betweenness centrality, five tissue types have observed means significantly greater than the expected means despite that all observed means were greater than the expected means (Figure 2b). For the closeness centrality, all but one (the lymph node) tissue types had an observed mean greater than expected: six tissue types had an observed mean significantly greater than expected while the remaining tissue types showed non-significant differences (Figure 2c and additional file 2, Table S2).

### Topological properties of the tissue-specific nodes

**Figure 3:**
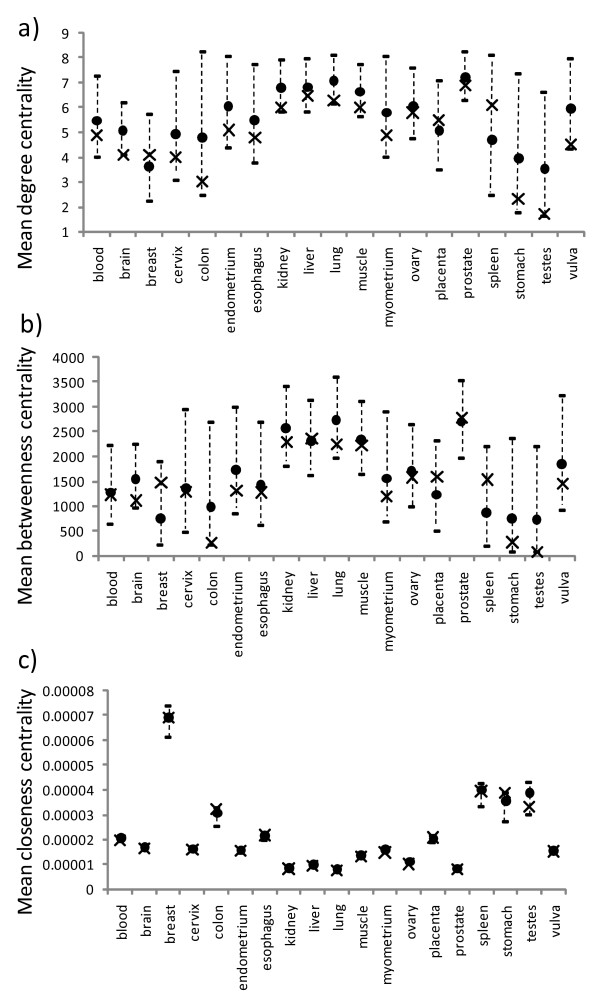
**Observed mean topological properties for the tissue-specific nodes in different PPI networks for the HuGE Index dataset**. The mean degree, betweenness, and closeness centralities are shown in a), b), and c), respectively. Each observed mean is shown as a cross and each expected mean by a circle, with its 95% confidence intervals shown as a vertical dotted line above and below the expected mean.

For the HuGE Index database, none of the tissue types had observed mean degree centralities for tissue-specific nodes significantly different from the expected means (Figure 3a). Three tissue types had observed means greater than the expected means, while the remaining tissue types had observed means lower than expected. For the betweenness centrality, the testes had an observed mean significantly lower than the expected mean, while the remaining tissue types showed non-significant differences (Figure 3b). For the closeness centrality, the ovary was the only tissue with an observed mean significantly lower than the expected mean, while the other tissue types had observed means not significantly different from expected (Figure 3c and additional file 2, Table S3).

**Figure 4:**
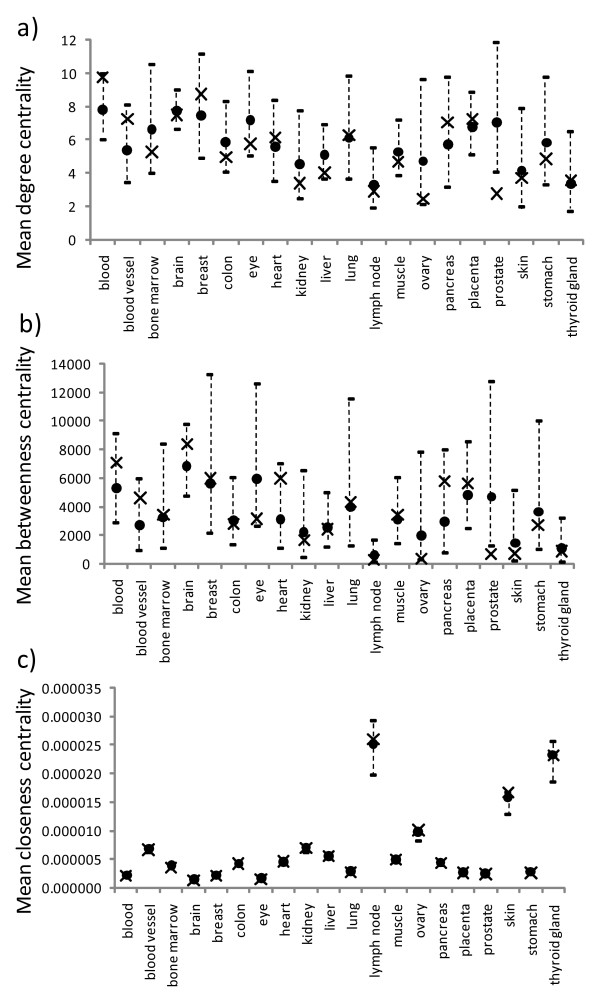
**Observed mean topological properties for the tissue-specific nodes in different PPI networks for the EST-SAGE dataset**. The mean degree, betweenness, and closeness centralities are shown in a), b), and c), respectively. Each observed mean is shown as a cross and each expected mean by a circle, with its 95% confidence intervals shown as a vertical dotted line above and below the expected mean.

For the EST-SAGE database, only the prostate has an observed mean degree centrality significantly lower than the expected mean, while the others had observed means not significantly different from expected (Figure 4a). For the betweenness centrality, the prostate again had an observed mean significantly lower than expected, while those for other tissue types were not significantly different from the expected means (Figure 4b). For the closeness centrality, all tissue types had observed means not significantly different from expected (Figure 4c and additional file 2, Table S4).

### Homophylic connectivity of the house-keeping nodes

**Figure 5:**
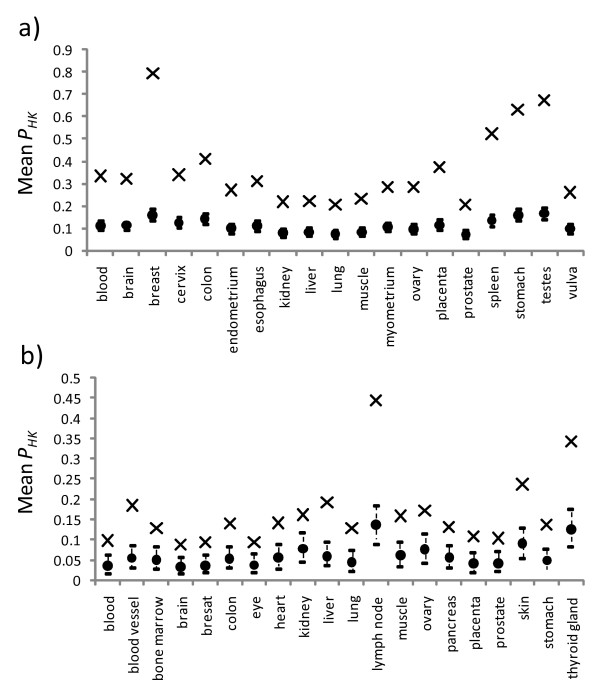
**Observed mean proportion of house-keeping neighbors for the house-keeping nodes in the different tissue types**. The results for the HuGE Index dataset are shown in a) and those for the EST-SAGE dataset in b). Each observed mean is shown as a cross and each expected mean by a circle, with its 95% confidence intervals shown as a vertical dotted line above and below the expected mean.

For each PPI network, we calculated the proportion of house-keeping neighbors for individual house-keeping nodes (*P*_*HK*_) and their mean. Figure 5a shows how the observed means compared with the model distribution for the 19 tissue types in the HuGE Index database. Our results show that the observed mean proportions were all significantly greater than the expected means, as they were all well above the upper limit of the 95% confidence interval of their corresponding model distributions. A similar pattern was seen for house-keeping nodes in the EST-SAGE dataset (Figure 5b).

### Homophylic connectivity of the tissue-specific nodes

**Figure 6:**
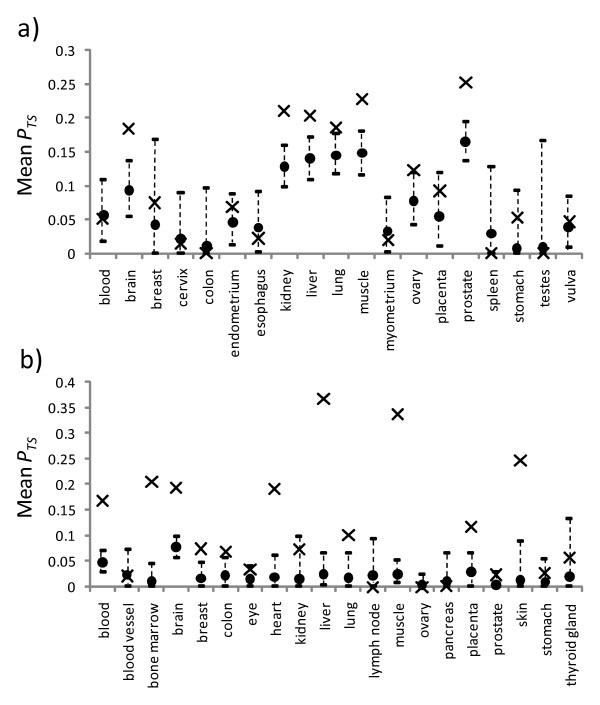
**Observed mean proportion of tissue-specific neighbors for the tissue-specific nodes in the different tissue types**. Results for the HuGE Index dataset are shown in a) while those for the EST-SAGE dataset are shown in b). Each observed mean is shown as a cross and each expected mean by a circle, with its 95% confidence intervals shown as a vertical dotted line above and below the expected mean.

For the HuGE Index dataset, 7 of the 19 tissue types had observed means significantly greater than the expected means, while the remaining tissue types showed a non-significant difference (Figure 6a). For the EST-SAGE dataset, 11 of the 20 tissue types had observed means significantly greater than the expected means, while the remaining tissue types showed a non-significant difference (Figure 6b). Note that, for both datasets, a few tissues had a mean *P*_*TS *_of 0 due to the fact that there were no connections between their tissue-specific nodes (e.g. the colon, spleen, and testes for the HuGE Index dataset and the lymph node and ovary for the EST-SAGE dataset).

## Discussion

In this paper, we analyzed the topological properties of proteins encoded by house-keeping genes and tissue-specific genes and their local connectivity structure in the PPI networks for a variety of human tissues. An interesting pattern in terms of how cells organize their inventory tools emerged. Although the results for the average degree and betweenness centrality for house-keeping nodes in a PPI network derived from the HuGE Index dataset were not statistically different from those for randomly selected nodes, in each of the tissue types examined, the proteins encoded by house-keeping genes tended to have a greater number of direct neighbors (i.e. a high degree centrality) and to occupy network positions that were incident to many shortest interaction paths (i.e. a high betweenness centrality) than randomly selected proteins in a PPI network. This finding was more evident in the analysis of the EST-SAGE dataset, where the results for several tissue types were statistically significant. Such a consistent observation across different tissue types and different gene expression platforms indicates that house-keeping genes tend to code for proteins of more topological importance in a PPI network. In contrast, the protein products of tissue-specific genes tended to occupy network positions no different from those of a group of randomly selected proteins. With relatively few exceptions, this was observed in most tissue types across both the HuGE Index and EST-SAGE datasets. Moreover, our results suggest that closeness centrality does not consistently reflect the topological importance of house-keeping genes in PPI networks. This is because the closeness centralities of a node and any of its direct neighbors should be similar, since there is only one link separating them [13]. Some house-keeping genes are bound to connect to some tissue-specific genes. Thus, if a house-keeping gene occupies a highly central position in a PPI network, as measured by closeness centrality, then its tissue-specific neighbors will also be important, and this results in many tissue-specific genes being more important than some house-keeping genes. The observation that house-keeping genes tend to occupy important network positions seems to fit the general trend that topologically important network positions tend to reflect common characteristics or vital processes in biology. For instance, proteins that have many interacting partners in a PPI network tend to be encoded by essential genes in yeast [10], and topologically important enzymes tend to be shared by many different bacterial species [13].

The second part of our analysis revealed that the protein products of house-keeping genes tended to connect or interact among themselves in a PPI network. Such homophylic connectivity was observed for all tissue types in the two databases used. However, homophylic connectivity of tissue-specific gene-encoded proteins was not so widely observed. If one pools the results from both datasets, then slightly fewer than half of the tissues examined exhibited homophylic connectivity for tissue-specific genes. Although house-keeping and tissue-specific functions are probably the two most fundamental biological functions in cell biology, we believe our findings still fit the general trend that network nodes performing similar biological functions tend to form clusters in a molecular network [23-26]. For instance, in metabolic and biochemical networks, metabolites tend to aggregate in the same network locations and form distinct functional modules or metabolic pathways [28,29].

The observation that house-keeping genes tended to be topologically important, whereas tissue-specific genes did not led us to speculate why nature has organized a cell's inventory tools in such a manner and to propose the following hypothesis. Imagine there is a hypothetical neutral cell, neutral in the sense that it is in an undifferentiated state. If the protein products of tissue-specific genes were located in topologically important positions in a PPI network such that they had many interacting partners, then it might be possible that the process of differentiation to a particular morphological state might involve other unnecessary tissue-specific genes. This could have two hypothetical drawbacks. First, expressing unnecessary and unrelated tissue-specific genes while performing tissue-specific functions or tasks is not an economical or efficient way for a neutral cell to utilize its resources when undergoing cell differentiation. Second, because of the expression of these unrelated tissue-specific genes, unwanted functions might be performed such that a neutral cell might fail to differentiate to the correct morphological state. In contrast, house-keeping proteins are topologically important because they are involved in processes that perform basic and common cell functions, without which cells of different types would have difficulties in their maintenance. A close inspection of our results and PPI networks provides hints supporting our hypothesis. For instance, beta actin is one of the house-keeping gene-encoded proteins that ranked high in terms of topological importance (within the 1^st ^percentile of the degree and betweenness distributions for all tissue types) in our PPI networks. It has many interacting neighbors, many of which, such as cofilin, gamma actin, profiling, and beta tubulin, are also encoded by house-keeping genes [30-33]. These proteins form the cytoskeleton that provides structural integrity to a cell and organizes cellular activities [3] and their expression in all cell types is therefore essential for a cell to function properly or even exist in the first place. One of the non-house-keeping neighbors of beta actin is troponin I [34], which combines with troponin T and troponin C to form the troponin complex [35] that plays an important role in the contraction of cardiac and skeletal muscles [3]. The constituent protein components of the troponin complex had average rankings of 688^th ^and 558^th ^in the degree and betweenness centralities, respectively, in the HuGE-derived muscle-specific PPI network, while the equivalent average rankings for the actin-cofilin-profilin-tubulin core were 279^th ^and 334^th^. As for the EST-SAGE-derived muscle-specific PPI network, the troponin complex components had average rankings of 736^th ^and 537^th ^in the degree and betweenness centralities, respectively, while the actin-cofilin-profilin-tubulin core was ranked 170^th ^and 158^th^. Thus, the troponin complex is, on average, of lower topological importance and is attached to the actin-cofilin-profilin-tubulin core at a more peripheral position in the muscle-specific PPI network. Another example is neurogenesis, the process of formation of nerve tissue [36]. The CRMP (collapsin response mediator protein) family plays key roles in growth cone guidance during neural development [37,38], and four members of this family, CRMP1, CRMP2, CRMP3, and CRMP5, could be mapped to the brain-specific PPI network for the EST-SAGE dataset. CRMP1, CRMP2, CRMP3, and CRMP5 interact sequentially to form a complex [39,40], which connects to the actin-cofilin-profilin-tubulin core via CRMP2 and beta tubulin [41], as well as via CRMP1 and profilin [42]. In the brain-specific PPI network, the CRMP complex was ranked on average 1864^th ^and 1531^st ^in terms of the degree and betweenness centralities, respectively, while the corresponding values for the actin-cofilin-profilin-tubulin core were 356^th ^and 294^th^. Again, this demonstrates that the actin-cofilin-profilin-tubulin core is located in a topologically important position in the PPI network, while the tissue-specific proteins, such as the CRMP complex, are more peripheral.

## Conclusion

In this paper, we have shown how a cell organizes its house-keeping and tissue-specific tools in a PPI network. Both house-keeping and tissue-specific functions are very broad functional categories and structural patterns in network organization have been observed. In general, our findings suggest that house-keeping genes are topologically important in a PPI network, whereas tissue-specific genes are not, and that both sets of protein products exhibit a tendency, although to different extents, to homophylic connectivity. Our findings led us to hypothesize that house-keeping genes tend to code for proteins that form the core of a PPI network, while tissue-specific genes are responsible for those at more peripheral positions of the network. The next challenge is to propose and explain the evolutionary mechanism that gave rise to the observed network organization of cellular tools.

## Methods

### Human Gene Expression Index

The Human Gene Expression Index (HuGE Index) [4] is a publicly available resource  which serves as a compendium of gene expression in normal human tissues. It contains gene expression patterns for 19 different tissue types analyzed using oligonucleotide microarrays. Each of these 19 tissue types was derived from a different human organ. For simplicity, we identify here each tissue-type by the name of the organ which the tissue was a part of. Genes that were expressed in at least one sample of each tissue type are defined as house-keeping genes; and a two-tailed *t*-test at the 99.99% confidence level was used to select tissue-specific genes after comparing gene expression profiles across different tissue types [4]. The HuGE Index database provides a list of expressed genes and a list of tissue-specific genes for each tissue type, together with a list of house-keeping genes expressed in these 19 tissues types. This gene list constitutes one of the two datasets used in this study and is referred to as the HuGE Index dataset.

### Expressed Sequence Tag and Serial Analysis of Gene Expression

In contrast to the microarray-based methodology used in the HuGE Index database, gene expression can also be analyzed using the tag-based techniques. Gene expression can be quantified using the Expressed Sequence Tag (EST) [7], and Pao et al. [9] have used the AC-test [43] to detect tissue-specific genes from EST gene expression profiles for a variety of tissue types. Gene expression can also be quantified using Serial Analysis of Gene Expression (SAGE) [8]; and, more recently, following the method of Pao et al. [9], Wang and Hwang used the AC-test to identify tissue-specific genes from SAGE profiles for several tissue types (our unpublished data). For each of the tissue types examined, we compiled a list of genes expressed in both gene expression platforms and refer to this as the EST-SAGE dataset. This dataset contains 20 different tissue types, and we identify each tissue-type by the name of the organ from which the tissue was derived. For this dataset, a gene is identified as a house-keeping gene if it is expressed in every tissue type and as a tissue-specific gene if, and only if, it is identified as a tissue-specific gene in both EST and SAGE platforms with a *p *value cut-off threshold of 10^-6 ^[9].

### Protein-protein interaction networks

The Human Protein Reference Database (HPRD) [27] contains information on pair-wise protein-protein physical interactions. We mapped genes from the HuGE Index and EST-SAGE datasets to their protein products in the HPRD. Genes for which the protein products could not be found in the HPRD or did not have interacting partners were excluded from analysis. Note that such exclusions resulted in different network sizes and in variation in the proportion of house-keeping gene-encoded and tissue-specific gene-encoded proteins in different tissue types. Furthermore, such a filtering process could also omit house-keeping genes whose protein products have no interaction partners in the HPRD for some tissue types; this then in turn resulted in unequal numbers of house-keeping genes among different tissue types in our study here. For the HuGE Index dataset, this mapping procedure created 19 PPI networks, each for a particular tissue type. Similarly, for the EST-SAGE dataset, the 20 different tissue types resulted in 20 different PPI networks. Here, we define clearly that a node in a PPI network represents a protein, and we call nodes representing proteins encoded by house-keeping genes house-keeping nodes and those representing proteins encoded by tissue-specific genes tissue-specific nodes. In all the PPI networks constructed, we ignored link directions between nodes. Because of the different numbers of genes in the different tissue types, the PPI networks constructed in this study were all of different sizes.

### Network fragmentation

We define a network fragment as a portion or a component of a network that consists of nodes that are only reachable from nodes in the same fragment. We further define the size of a network fragment as the number of nodes it contains. For every PPI network constructed in this study, we determined the number of network fragments and their respective sizes in order to gain insights into the connectivity of a PPI network.

### Percentage of overlap between PPI networks derived from the HuGE and EST-SAGE datasets

The HuGE and EST-SAGE datasets have some tissue types in common. For each of those common tissue types (or PPI networks), we determined the number of genes (or nodes) and the number of house-keeping or tissue-specific genes (or nodes) that appeared in both datasets. For each common tissue type, we then determined the proportion of common nodes in the total number of nodes in a PPI network for a given dataset, as well as the proportion of common house-keeping and tissue-specific nodes in the total number of nodes in the same database. These proportions can be regarded as the percentage of overlap between the HuGE and EST-SAGE datasets for a common tissue type.

### Measures of topological importance

The topological importance of a node in a network measures how prominent this particular node is to others in the same network. It is also a measure of how central the position of a node is in relation to others in the same network. A node might be topologically important simply because it has many connecting neighbors or occupies a network position that is close to all other nodes. There exist several network indices that can quantify different aspects of topological importance for all nodes in a network. Here, we used three well-known indices that measure the topological importance or centrality of nodes in a network [44,45], and calculated them by using UCINET [46]. The degree centrality (*D*_*i*_) is simply the number of direct neighbors of a given node *i *and is a local measure of positional importance. A node with a high degree centrality is important, since it has many direct interacting partners. The betweenness centrality of a node *i *(*B*_*i*_) measures how frequently node *i *is incident to all shortest paths in a network [44]:

$$B_{i}=\sum_{j,k=1,j<k}^{N}g_{jk}(i)/g_{jk},$$

where *i *≠ *j *and *k*; *N *is the number of nodes in the network; *g*_*jk *_is the number of shortest paths between nodes *j *and *k*, and *g*_*jk*_(*i*) is the number of these shortest paths to which node *i *is incident. A shortest path between a node pair is a path with the minimum number of links when one travels from one node to the other. In the above formulation *g*_*jk*_/*g*_*jk*_(*i*) is the probability of node *i *being on the shortest paths between connected node pair *j *and *k *(i.e. *j *and *k *can reach each other); and betweenness centrality of a node *i *is therefore the sum of those probabilities with *jk *covering all connected node pairs excluding node *i *itself (hence the restriction *i *≠ *j *and *k*) and a node pair can only be counted once (since node pairs *jk *and *kj *are the same, the restriction *j *<*k *in the summation term is necessary). Note that only connected node pairs with existing or finite shortest paths are considered in the calculation of betweenness centrality, therefore betweenness centrality can be computed even for fragmented networks [44]. The betweenness centrality is a non-local measure of topological importance. A node with a high betweenness centrality is important, as it participates in, or mediates, many indirect interactions between any other two nodes. Lastly, the closeness centrality of a node *i *(*C*_*i*_) is a distance-based measure [44]:

$$C_{i}={\left[\sum_{j=1,i\neq j}^{N}d_{ij}\right]}^{-1},$$

where *d*_*ij *_is the length of the shortest path (distance) between nodes *i *and *j*, and *N *is the number of nodes in the network. Closeness centrality of a node *i *is simply the inverse of the sum of the lengths of shortest paths *d*_*ij *_between *i *and all other nodes in the same network (i.e. let *j *be a node other than *i *itself, then *j *covers all nodes from 1 to *N *excluding *i *= *j *when calculating *C*_*i*_). Closeness centrality simply measures how close a node is to all others in the same network and is also a non-local measure of positional importance, since all nodes in the network are taken into account when evaluating a node's closeness centrality. If a node is very close to other nodes in the same network, then its closeness centrality will be large. A node with a large closeness centrality is important, as it can affect others rapidly and can also be rapidly affected by others. Another network index closely related to closeness centrality is the farness of a node, which is simply the inverse of its closeness centrality (i.e. the sum of the lengths of shortest paths between a given node and all others in the same network). Thus, both closeness centrality and farness of a node contain the same information about its position in a network. Unlike betweenness centrality, the calculation of closeness centrality (or farness) of a node requires it being reachable from all others in the same network; in other words the network must not be fragmented and all shortest distance must be finite. Fortunately such a shortcoming can be remedied by substituting the infinite distance with the theoretical maximum distance *N *during the calculation of closeness centrality or farness of a node by using UCINET [46].

### Testing the topological importance of house-keeping and tissue-specific nodes

We used the above network nodal indices to measure the topological importance of individual nodes for each PPI network in this study, therefore a node's importance can be quantified from three different perspectives. For each of these topological measures, we carried out the following test to investigate whether house-keeping nodes occupied important network positions in a PPI network. First, we calculated, for each PPI network, the observed mean topological importance of the house-keeping nodes. Assuming that there are *N*_*HK *_house-keeping nodes in a PPI network, we then randomly sampled *N*_*HK *_nodes to be our new house-keeping nodes and determined their mean topological importance. Repeating this sampling process 1000 times generated a model distribution of means against which the significance of our observed means could be tested. The expected value of the mean topological importance of house-keeping nodes is the average of this model distribution. The 95% confidence interval of the mean topological importance of house-keeping nodes can also be defined from the model distribution, as 2.5% of the total number of simulations produce means greater than the upper limit of the interval and 2.5% means lower than the lower limit. For the degree, betweenness and closeness centralities, if the house-keeping nodes do indeed on average occupy important positions in a PPI network, then the means of their degree, betweenness and closeness centralities should be greater than the upper bounds of the corresponding 95% confidence interval. The topological importance of tissue-specific nodes was tested using the same methodology.

### Homophylic connectivity of house-keeping nodes and tissue-specific nodes

We defined the homophylic connectivity of house-keeping nodes as the tendency for house-keeping nodes to connect to house-keeping nodes in a PPI network. For each PPI network, we determined, for each house-keeping node *i*, the number of its direct neighbors (i.e. *D*_*i*_, the degree centrality) and counted how many of these were house-keeping nodes (*D*_*HK*, *i*_); we then defined *P*_*HK*, *i *_as the proportion of these direct neighbors that were house-keeping nodes:

*P*_*HK*, *i *_= *D*_*HK*, *i*_*/(D*_*i*_).

Likewise, the homophylic connectivity of tissue-specific nodes is the tendency for them to connect among themselves in a PPI network. Similarly, for a tissue-specific node *i*, the proportion of its direct neighbors that are tissue-specific nodes is:

*P*_*TS*, *i *_= *D*_*TS*, *i*_*/(D*_*i*_),

where (*D*_*TS*, *i*_) is the number of its tissue-specific neighbors.

### Testing the homophylic connectivity of house-keeping nodes and tissue-specific nodes

To test the significance of the homophylic connectivity of house-keeping nodes in a given PPI network, we first calculated the observed mean *P*_*HK *_(we have dropped the subscript *i *for simplicity), then we constructed a random network of the same size and degree distribution as the original PPI network. Such a randomization process results in each node having the same number of interacting neighbors as the original PPI network, and the only change is the identity of its direct neighbors. We then calculated *P*_*HK *_for all house-keeping nodes and determined the mean *P*_*HK*_. Generating 1000 random networks gave a model distribution of the mean *P*_*HK*_. The expected value of the mean *P*_*HK *_is the average of this model distribution, and its 95% confidence interval can be determined in the same way as that for the mean topological importance mentioned above. If the house-keeping nodes show a tendency towards homophylic connectivity, then the observed mean *P*_*HK *_should be greater than the upper limit of the 95% confidence interval of the model mean. The significance of the homophylic connectivity for tissue-specific nodes in a given PPI network was also tested using this method.

## Authors' contributions

WHL, WCL and MJH conceived the study; WHL extracted data and constructed the PPI networks; WHL carried out the analysis; WHL, WCL and MJH prepared the manuscript. All authors read and approved the final manuscript.

## Supplementary Material

Additional file 1 **Network Data.** Network data used in this study including lists of house-keeping and tissue-specific nodes in each PPI network. Click here for file

Additional file 2 **Supplementary Tables.** Tables summarize the observed mean closeness centralities for house-keeping nodes and tissue-specific nodes and their expected means with 95% confidence intervals for each PPI network. Click here for file
