# Supplementary material for: Topological and organizational properties of the products of house-keeping and tissue-specific genes in protein-protein interaction networks
Source: BMC Syst Biol. 2009 Mar 11;3:32. doi: 10.1186/1752-0509-3-32 (PMC2663781; doi:10.1186/1752-0509-3-32)
Supplement: Additional file 2 — Supplementary Tables. Tables summarize the observed mean closeness centralities for house-keeping nodes and tissue-specific nodes and their expected means with 95% confidence intervals for each PPI network. [file 1752-0509-3-32-S2.doc]

Table S1: The mean closeness centralities for house-keeping nodes in HuGE Index-derived PPI networks.

|  | HK_mean | R_2.5% | R_mean | R_97.5% |
| --- | --- | --- | --- | --- |
| blood | 0.000020739 | 0.000019956 | 0.000020597 | 0.000021180 |
| brain | 0.000016849 | 0.000016373 | 0.000016859 | 0.000017301 |
| breast | 0.000068335 | 0.000067301 | 0.000069491 | 0.000071433 |
| cervix | 0.000017128 | 0.000016589 | 0.000017139 | 0.000017603 |
| colon | 0.000029737 | 0.000029720 | 0.000030669 | 0.000031552 |
| endometrium | 0.000015445 | 0.000015217 | 0.000015637 | 0.000016002 |
| esophagus | 0.000021081 | 0.000020852 | 0.000021460 | 0.000021990 |
| kidney | 0.0000086067 | 0.0000083087 | 0.0000085283 | 0.0000087213 |
| liver | 0.0000098104 | 0.0000095637 | 0.0000098153 | 0.000010016 |
| lung | 0.0000078696 | 0.0000076652 | 0.0000078565 | 0.0000080219 |
| muscle | 0.000013410 | 0.000013185 | 0.000013475 | 0.000013739 |
| myometrium | 0.000015860 | 0.000015524 | 0.000015976 | 0.000016367 |
| ovary | 0.000010929 | 0.000010637 | 0.000010976 | 0.000011273 |
| placenta | 0.000020618 | 0.000019794 | 0.000020434 | 0.000021045 |
| prostate | 0.0000081925 | 0.0000079899 | 0.0000081862 | 0.0000083520 |
| spleen | 0.000039479 | 0.000038885 | 0.000040108 | 0.000041265 |
| stomach | 0.000035188 | 0.000034281 | 0.000035557 | 0.000036761 |
| testes | 0.000039206 | 0.000037415 | 0.000038990 | 0.000040466 |
| vulva | 0.000015602 | 0.000015121 | 0.000015534 | 0.000015863 |

The table summarizes the observed mean closeness centralities for house-keeping nodes (HK_mean) in different PPI networks for the HuGE dataset; also included are their expected means (R_mean) and 95% confidence intervals (R_2.5% and R_97.5% are the lower and upper bounds of the interval respectively).

Table S2: The mean closeness centralities for house-keeping nodes in EST-SAGE-derived PPI networks.

|  | HK_mean | R_2.5% | R_mean | R_97.5% |
| --- | --- | --- | --- | --- |
| blood | 0.0000021957 | 0.0000021067 | 0.0000021603 | 0.0000022038 |
| blood vessel | 0.0000069538 | 0.0000065970 | 0.0000067964 | 0.0000069829 |
| bone marrow | 0.0000039023 | 0.0000037265 | 0.0000038525 | 0.0000039630 |
| brain | 0.0000014157 | 0.0000013528 | 0.0000013876 | 0.0000014167 |
| breast | 0.0000021514 | 0.0000020609 | 0.0000021111 | 0.0000021503 |
| colon | 0.0000043145 | 0.0000040230 | 0.0000041625 | 0.0000042866 |
| eye | 0.0000016024 | 0.0000015251 | 0.0000015632 | 0.0000015948 |
| heart | 0.0000046276 | 0.0000043571 | 0.0000044971 | 0.0000046214 |
| kidney | 0.0000072898 | 0.0000067736 | 0.0000070230 | 0.0000072430 |
| liver | 0.0000056502 | 0.0000053439 | 0.0000055405 | 0.0000057082 |
| lung | 0.0000029368 | 0.0000028037 | 0.0000028881 | 0.0000029642 |
| lymph node | 0.000024394 | 0.000023625 | 0.000025213 | 0.000026656 |
| muscle | 0.0000049084 | 0.0000046673 | 0.0000048206 | 0.0000049542 |
| ovary | 0.0000098959 | 0.0000094110 | 0.0000097293 | 0.000010014 |
| pancreas | 0.0000045285 | 0.0000042532 | 0.0000043944 | 0.0000045224 |
| placenta | 0.0000026804 | 0.0000025668 | 0.0000026347 | 0.0000026901 |
| prostate | 0.0000025141 | 0.0000024001 | 0.0000024639 | 0.0000025219 |
| skin | 0.000015863 | 0.000015047 | 0.000015692 | 0.000016291 |
| stomach | 0.0000028220 | 0.0000026640 | 0.0000027506 | 0.0000028332 |
| thyroid gland | 0.000023553 | 0.000022054 | 0.000023229 | 0.000024302 |

The table summarizes the observed mean closeness centralities for house-keeping nodes (HK_mean) in different PPI networks for the EST-SAGE dataset; also included are their expected means (R_mean) and 95% confidence intervals (R_2.5% and R_97.5% are the lower and upper bounds of the interval respectively).

Table S3: The mean closeness centralities for tissue-specific nodes in HuGE Index-derived PPI networks.

|  | TS_mean | R_2.5% | R_mean | R_97.5% |
| --- | --- | --- | --- | --- |
| blood | 0.000019943 | 0.000019268 | 0.000020592 | 0.000021647 |
| brain | 0.000016693 | 0.000016247 | 0.000016861 | 0.000017397 |
| breast | 0.000068986 | 0.000061424 | 0.000069424 | 0.000073754 |
| cervix | 0.000016122 | 0.000016122 | 0.000016122 | 0.000016122 |
| colon | 0.000032483 | 0.000025527 | 0.000030670 | 0.000032684 |
| endometrium | 0.000015722 | 0.000014811 | 0.000015638 | 0.000016254 |
| esophagus | 0.000021841 | 0.000020026 | 0.000021472 | 0.000022381 |
| kidney | 0.0000084509 | 0.0000083577 | 0.0000085293 | 0.0000086888 |
| liver | 0.0000097015 | 0.0000095970 | 0.0000098173 | 0.000010001 |
| lung | 0.0000078256 | 0.0000077086 | 0.0000078560 | 0.0000079805 |
| muscle | 0.000013341 | 0.000013236 | 0.000013475 | 0.000013705 |
| myometrium | 0.000014967 | 0.000014954 | 0.000015986 | 0.0000167123 |
| ovary | 0.000010250 | 0.000010532 | 0.000010979 | 0.000011363 |
| placenta | 0.000020989 | 0.000019151 | 0.000020441 | 0.000021539 |
| prostate | 0.0000081294 | 0.0000080394 | 0.0000081852 | 0.0000083123 |
| spleen | 0.000039608 | 0.000033340 | 0.000040087 | 0.000042710 |
| stomach | 0.000038820 | 0.000027122 | 0.000035607 | 0.000038972 |
| testes | 0.000033253 | 0.000030026 | 0.000038977 | 0.000043067 |
| vulva | 0.000015348 | 0.000014831 | 0.000015535 | 0.000016097 |

The table summarizes the observed mean closeness centralities for tissue-specific nodes (TS_mean) in different PPI networks for the HuGE dataset; also included are their expected means (R_mean) and 95% confidence intervals (R_2.5% and R_97.5% are the lower and upper bounds of the interval respectively).

Table S4: The mean closeness centralities for tissue-specific nodes in EST-SAGE-derived PPI networks.

| tissue | TS_mean | R_2.5% | R_mean | R_97.5% |
| --- | --- | --- | --- | --- |
| blood | 0.0000022189 | 0.0000020867 | 0.0000021606 | 0.0000022163 |
| blood vessel | 0.0000067048 | 0.0000062944 | 0.0000067967 | 0.0000071084 |
| bone marrow | 0.0000036490 | 0.0000035321 | 0.0000038495 | 0.0000040342 |
| brain | 0.0000014056 | 0.0000013637 | 0.0000013880 | 0.0000014096 |
| breast | 0.0000021781 | 0.0000019963 | 0.0000021119 | 0.0000021783 |
| colon | 0.0000043036 | 0.0000039143 | 0.0000041627 | 0.0000043783 |
| eye | 0.0000015534 | 0.0000014875 | 0.0000015627 | 0.0000016216 |
| heart | 0.0000047161 | 0.0000041676 | 0.0000044945 | 0.0000047222 |
| kidney | 0.0000070732 | 0.0000062417 | 0.0000070261 | 0.0000075023 |
| liver | 0.0000056561 | 0.0000051688 | 0.0000055378 | 0.0000058667 |
| lung | 0.0000028252 | 0.0000026376 | 0.0000028872 | 0.0000030254 |
| lymph node | 0.000026000 | 0.000019715 | 0.000025183 | 0.000029287 |
| muscle | 0.0000050517 | 0.0000045376 | 0.0000048204 | 0.0000050523 |
| ovary | 0.000010191 | 0.0000082810 | 0.0000097321 | 0.000010290 |
| pancreas | 0.0000044004 | 0.0000039822 | 0.0000043895 | 0.0000046250 |
| placenta | 0.0000026756 | 0.0000025205 | 0.0000026349 | 0.0000027032 |
| prostate | 0.0000024650 | 0.0000022905 | 0.0000024652 | 0.0000025591 |
| skin | 0.000016719 | 0.000012793 | 0.000015688 | 0.000016736 |
| stomach | 0.0000026753 | 0.0000024483 | 0.0000027504 | 0.0000029077 |
| thyroid gland | 0.000023246 | 0.000018570 | 0.000023223 | 0.000025666 |

The table summarizes the observed mean closeness centralities for tissue-specific nodes (TS_mean) in different PPI networks for the EST-SAGE dataset; also included are their expected means (R_mean) and 95% confidence intervals (R_2.5% and R_97.5% are the lower and upper bounds of the interval respectively).
